# Corkscrew Seals: Grey Seal (*Halichoerus grypus*) Infanticide and Cannibalism May Indicate the Cause of Spiral Lacerations in Seals

**DOI:** 10.1371/journal.pone.0156464

**Published:** 2016-06-02

**Authors:** Andrew Brownlow, Joseph Onoufriou, Amanda Bishop, Nicholas Davison, Dave Thompson

**Affiliations:** 1 Scottish Marine Animal Stranding Scheme, SAC Veterinary Services Drummondhill, Stratherrick Road, Inverness, IV2 4JZ, United Kingdom; 2 Sea Mammal Research Unit, University of St Andrews, St Andrews, Fife, KY16 8LB, United Kingdom; 3 Durham University, School of Biology and Biomedical Sciences, Durham, DH1 3LE, United Kingdom; Sonoma State University, UNITED STATES

## Abstract

Large numbers of dead seals with characteristic spiral lesions have been washing ashore around the North Atlantic over the past two decades. Interactions with ship propellers and shark predation have been suggested as the likely causal mechanisms. However, new evidence points towards a more likely candidate: grey seal predation. An adult male grey seal was observed and recorded catching, killing and eating five weaned grey seal pups over a period of one week on the Isle of May, Scotland. A further 9 carcasses found in the same area exhibited similar injuries. Post mortem analysis of lesions indicated the wound characteristics were similar to each other and in 12 of the 14 carcasses analysed, were indistinguishable from carcasses previously attributed to propeller interaction. We therefore propose that most of the seal carcasses displaying spiral lacerations in the UK are caused by grey seal predation. Cases in other locations should be re-evaluated using the scoring system presented here to identify whether grey seal predation is a major cause of mortality in phocid seals.

## Introduction

Grey seals (*Halichoerus grypus*) and harbour seals (*Phoca vitulina*) are sympatric predators throughout much of their distribution in the Northern Atlantic. In some areas of Scotland, where approximately 30% of the European harbour seal population is found, harbour seals are in steep decline [1]. Since 1985, carcasses of grey seals (*Halichoerus grypus*) and harbour seals (*Phoca vitulina)* with dramatic and characteristic spiral lacerations have been reported stranded at various locations around the UK coast. These reports increased annually to a sustained high level since 2009 [2,3]. Similar injuries on harp (*Phoca groenlandica*) and hooded (*Cystophora cristata*) seals, along with grey and harbour seals, have been observed at various other locations around the North Atlantic with particularly large numbers recorded in Canada since the early 1990’s [4,5]. Reported strandings followed a consistent seasonal pattern throughout the range, with harbour seal strandings restricted to the spring and summer months, and grey seal strandings peaking during the autumn and winter [2,3].

The regular and consistent nature of the wounds in the UK were thought to stem from an anthropogenic rather than a natural cause [3,6]. The characteristic spiral or ‘corkscrew’ wound was thought to be caused by mechanical contact with a blade rotating within a duct [2,3,6]. Spatial and temporal patterns of strandings supported the theory that ducted propellers on ships were the most plausible explanation for the characteristic wound pattern observed around the UK [2,3]. However, other mechanisms such as shark predation [4] and more recently, with regards to phocid deaths in Germany and Canada, grey seal predation [7,8] have been suggested.

In Scotland the need to identify a cause of these injuries is high given the unexplained decline of several harbour seal populations [6]. Rapid declines have been recorded since 2000 in south-east and north-east Scotland [9], in areas where a large proportion of the harbour seal corkscrew cases have been reported. In particular, the population of harbour seals in the Tay and Eden Special Area of Conservation has declined by more than 90% over the past 15 years to an estimated 29 individuals in a 2014 census [10]. It is unclear whether these corkscrew events were the initial cause of the decline but the consistent numbers being removed annually through corkscrew lacerations (32 between 2008 and 2014) are unsustainable. In this region the annual incidence of spiral lesions has remained reasonably constant, however the rapid population decline means that the resulting per capita mortality rate is increasing.

In addition to harbour seal mortality, concentrations of grey seal pup carcasses with spiral injuries have also been noted in several areas of the UK. The Isle of May, in SE Scotland, has been a hotspot for spiral injuries with multiple cases recorded in 2010, 2012, 2013 and 2014. Here we report detailed observations of a series of events of conspecific aggression, infanticide and cannibalism by an adult male grey seal on the Isle of May breeding colony in December 2014. We also describe the results from post mortem examination of recently weaned pups from the same location which were unlikely to have left land prior to death. We then compare these observations to previous corkscrew seal records and relate them to recent observations of attacks on other seals by adult male grey seals.

## Materials and Methods

All international, national and institutional guidelines for the care and use of use of animals were followed in this study. All observations conformed to the UK Animals (Scientific Procedures Act, 1986). Permission to use the Isle of May for scientific investigations was given by Scottish Natural Heritage.

This study was carried out between November 2014 and January 2015 on the Isle of May, a 0.5 km^2^ island in the Firth of Forth, Scotland. It is the largest grey seal breeding colony on the east coast of Scotland, producing over 2000 pups between late October and early December each year. During breeding season the population is dispersed predominantly towards the southern and northern extremes of the island. Females arrive earlier in the season to pup and males arrive later to begin copulation. Females suckle their pups for approximately 18 days and then abandon them on the island [11]. Weaned pups then remain on the breeding colony fasting for between 10 and 40 days [12,13]. The observations described below were carried out after the peak weaning date, i.e. towards the end of the breeding season when the colony consisted mainly of weaned pups rather than mother-pup pairs and when a greater number of adult males were hauled out.

### Visual observations

An initial predation event by an adult male grey seal on a weaned pup was observed and video recorded incidentally during a behavioural observation study on the Isle of May (details presented in results). The adult male was then monitored continuously during daylight hours and all subsequent attacks were video recorded.

Four time lapse and two video cameras were setup to monitor the seal’s behaviour. The systems focussed on the known location of predation events, the breeding area where the male was observed to remain (approximately 0.015 km^2^) and an area to the north of the breeding site where corkscrew carcasses had been recovered (approximately 0.03 km^2^). In addition, an observer focussed on the study male throughout daylight hours to provide photographs and video records of any predation events.

### Post mortem examination

The total area of the Isle of May is approximately 0.57 km^2^[14] and a total of 14 pups were discovered with traumatic lesions within a 0.02 km^2^ area. Of these, 11 were retrieved for examination with the remaining 3 cases being assessed from digital photographs. Cases were evaluated for the presence or absence of specific pathological attributes using a modified version of a scoring scheme devised to identifying cork screw injuries in previous studies. All cases were examined and scored by the same veterinary pathologist with several years’ experience leading pinniped necropsies.

Cases were assessed for evidence of each of the attributes detailed in Table 1. A score of -1, 0 or 1 was allocated to indicate the absence, insufficient data to identify or presence of an attribute respectively.

**Table 1:** Pathological attributes of the weaned grey seal pup carcasses found on the Isle Of May in December 2014.

ID	*M410/14*	*M411/14*	*M416/14*	*M432/14*	*M431/14*	*M387/14*	*M373/14*	*M413/14*	*M414/14*	*M417/14*	*M433/14*	*M415/14*	*M412/14*	*M409/14*
Date found	03-Dec	03-Dec	04-Dec	08-Dec	06-Dec	02-Dec	28-Nov	03-Dec	03-Dec	05-Dec	09-Dec	03-Dec	03-Dec	03-Dec
Sex	F	M	M	F	M	M	M	F	F	M	F	F	M	F
**Axial girth at necropsy (cm)**	70	66	91	48	70	52	57	91	N/A	83	75	98	87	93
**Location (TC tidal channel FW freshwater pool)**	TC	TC	FW	FW	FW	FW	TC	FW	FW	FW	TC	FW	TC	TC
**Attack observed?**	NO	NO	YES	YES	NO	YES	NO	NO	YES	YES	NO	NO	NO	NO
**Necropsied?**	YES	YES	YES	NO	NO	YES	YES	YES	YES	YES	NO	YES	YES	YES
**Significant areas of skin or tissue missing**	**1**	**1**	**1**	**1**	**1**	**1**	**1**	**1**	**1**	**1**	**1**	**1**	**-1**	**1**
**Undermining of blubber**	**1**	**1**	**1**	**1**	**1**	**1**	**1**	**1**	**1**	**1**	**1**	**1**	**-1**	**-1**
**Rakemarks in blubber**	**1**	**1**	**1**	**1**	**1**	**1**	**1**	**1**	**1**	**1**	**-1**	**1**	**-1**	**-1**
**Smooth wound margin**	**1**	**1**	**1**	**1**	**1**	**1**	**1**	**1**	**-1**	**1**	**1**	**-1**	**-1**	**-1**
**Single linear lesion (one or more rotations)**	**1**	**1**	**1**	**1**	**1**	**1**	**1**	**1**	**-1**	**-1**	**1**	**-1**	**-1**	**-1**
**Ragged wound margin**	**-1**	**-1**	**1**	**-1**	**1**	**1**	**-1**	**1**	**1**	**1**	**-1**	**1**	**1**	**1**
**Evidence of any skeletal trauma**	**1**	**1**	**1**	**1**	**1**	**1**	**-1**	**-1**	**1**	**1**	**-1**	**-1**	**1**	**-1**
**Avulsion of one or both scapula**	**1**	**1**	**1**	**1**	**-1**	**-1**	**1**	**1**	**1**	**1**	**-1**	**1**	**-1**	**-1**
**Punctate lesions elsewhere**	**-1**	**-1**	**1**	**1**	**1**	**1**	**-1**	**1**	**1**	**-1**	**1**	**1**	**1**	**-1**
**Lesion begins at mouth**	**1**	**1**	**-1**	**1**	**1**	**-1**	**-1**	**-1**	**-1**	**-1**	**1**	**-1**	**1**	**1**
**Punctate lesions on muzzle**	**1**	**1**	**-1**	**-1**	**-1**	**-1**	**1**	**-1**	**-1**	**-1**	**1**	**-1**	**1**	**1**
**Skeletal trauma to scapula**	**1**	**1**	**1**	**1**	**-1**	**-1**	**1**	**-1**	**-1**	**-1**	**-1**	**-1**	**-1**	**-1**
**Skeletal trauma to head**	**1**	**1**	**-1**	**-1**	**-1**	**-1**	**-1**	**-1**	**-1**	**-1**	**-1**	**-1**	**1**	**1**
**Assessment for grey seal predation?**	Likely	Likely	Definite	Definite	Likely	Definite	Likely	Likely	Definite	Definite	Likely	Likely	Likely	Likely
**Assessment as a ‘corkscrew’ seal case**	Typical	Typical	Similar	Typical	Similar	Typical	Typical	Similar	Atypical	Similar	Typical	Atypical	Atypical	Atypical

A pathological assessment was made to determine the likelihood that the cause of death was due to seal predation and whether the injuries were similar to the archetypal pattern of spiral or ‘corkscrew’ lesions as reported in Bexton *et al*. (2012) and Onoufriou *et al*. (2014). The likelihood scores were as follows:

- Definite: Cases observed as being predated by a grey seal
- Likely: Morphological changes most consistently explained by seal predation.
- Possible: Morphological features consistent with predation but other causes of death plausible.
- Unlikely: Morphological features more consistent with other causes of death.

Cases were further assessed for the similarity of the lesion pattern to cases previously attributed as spiral or ‘corkscrew’ lesions

- Archetypal: Cases indistinguishable from a spiral trauma lesion pattern and only these pathological attributes were noted. (Attributes detailed in Bexton *et al*. 2012).
- Typical: Cases present with many attributes seen in spiral trauma cases, specifically a single, smooth edged spiral wound extending one or more rotations around the body, but additional lesion morphology, not seen in archetypal cases, was noted.
- Similar: Cases exhibit some attributes seen in spiral trauma cases however significant other lesion morphology, notably ragged edges to wound margins, was noted.
- Atypical: Lesions do not match spiral trauma cases

## Results

### Observations of infanticide and cannibalism by an adult male grey seal

**Fig 1:**
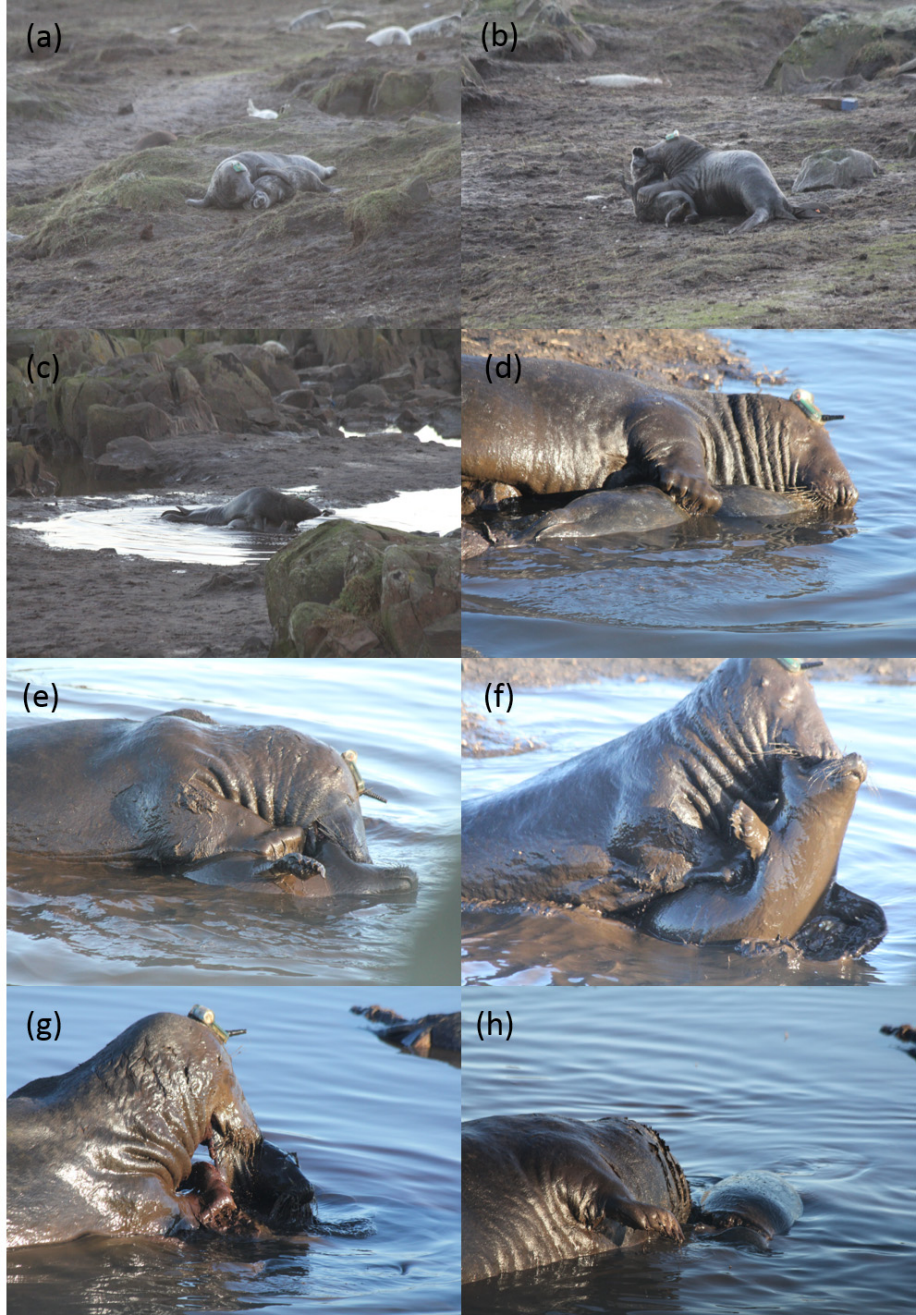
The study male (a) capturing a weaned pup, (b) lifting and dragging the pup towards the freshwater pool, (c) and (d) forcing the pup under the water to subdue it, (e) clamping his jaw around the scruff of the pups neck while locking his fore-flippers to the mid-section, (f) pulling upwards with his jaw while pushing downwards with his fore-flippers, (g) tearing flesh from the carcass which now displays an open wound and (h) resting after feeding on the pup which now displays a spiral laceration or ‘corkscrew cut’.

On the 2^nd^ December 2014 an adult male grey seal was seen catching a weaned grey seal pup on the Isle of May. The pup was presumed weaned due to its close proximity to other pups of similar age and the absence of adult females without pups in the immediate vicinity. It held the pup by the scruff of the neck and dragged it to a shallow freshwater pool (Fig 1). The adult then climbed on top of the pup, forced its head under water and held it until its struggles subsided.

**Fig 2:**
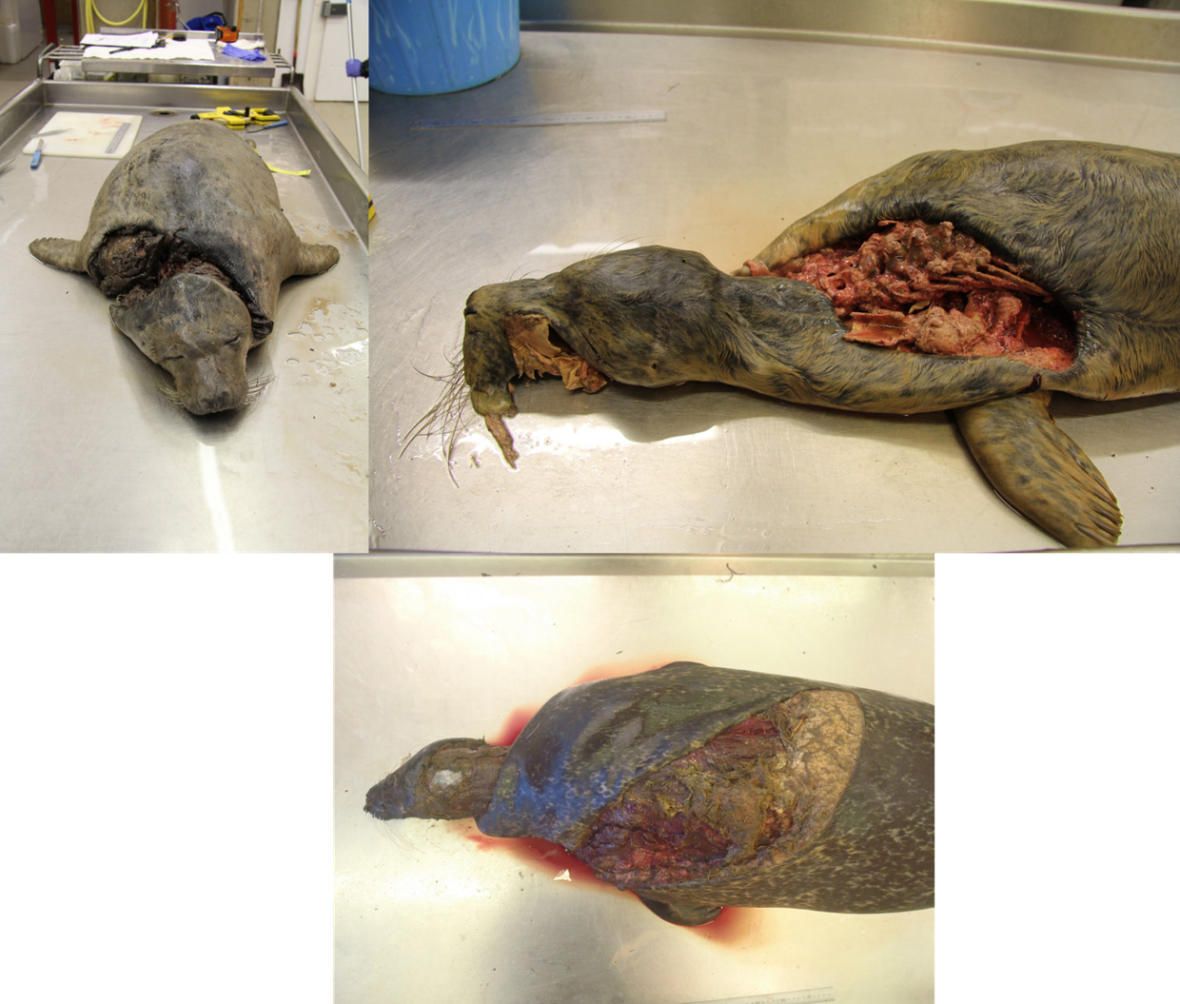
(top-left) A recently weaned grey seal pup found on the Isle of May in December 2014. The spiral laceration wound had been inflicted by an adult male grey seal; (top-right) a recently weaned grey seal pup found on the Isle of May in December 2014. The wounds were not witnessed being inflicted but were similar to those found on individuals cannibalised by the adult male grey seal; (bottom) a pregnant female harbour seal found on the Fife coast, North of the Isle of May in January 2014. These wounds were assumed to be propeller damage and are similar to wounds found on individuals cannibalised by the adult male grey seal.

The male seal then proceeded to bite the back of the neck and simultaneously pull back with its head while pushing away with his fore flipper. This caused the skin to tear and caused the blubber layer along the line of the tear to detach from the underlying body musculature (Fig 2). The study male then forced his lower jaw under the lip of the tear, biting down on the skin and then pulling back from the wound before swallowing several small sections of blubber and skin.

The pup was seen to move independent of the actions of the adult male 12 minutes after being dragged to the pool. A significant amount of mud and silt was found throughout the bronchial tree indicating death was primarily due to drowning however hypovolemic shock due to blood loss would have also been a significant factor (*see below*).

The process from the capture event to the discarding of the carcass lasted approximately 41 minutes. When the adult male later moved out of the pool, the pup carcass was retrieved and transported to the mainland for necropsy.

Over the next six days the male was observed catching four more weaned pups and killing them using a similar method. This resulted in similar wounds (Table 1) and in each case the male ate a quantity of blubber. In total 5 events were video recorded and/or photographed. A detailed description of the behaviour displayed in these events is presented in Bishop *et al*. (*In press*) [15].

In addition, a further three pup carcasses with similar wounds were retrieved from the freshwater pool following attacks during the night which were not observed. Although cannibalism events were not directly observed in these cases the same adult male was seen resting in or near the pool next to the carcass on each occasion and no other adult seals were seen in that pool over the entire study period.

Over an 11 day period an additional six pup carcasses were retrieved from a tide channel (TC) adjacent to the freshwater pools. Four of these had similar wounds while two were found with severe injuries to the front of the skull. Table 1 presents details of the locations and timings of carcass discoveries.

All eight carcasses in the pool are assessed to have been killed by the same adult male seal. The traumatic cause of death was not witnessed for the six carcasses found in the tide channel. However, the first one was found on 28^th^ November, four days before the first observed predation event and the last was found on the morning of 9^th^ December having been killed during the previous night. The male left the island on the night of the 8^th^ December. All six carcasses were found within 90m of the freshwater pool, on the route that the male would have followed when moving between the freshwater pool and the sea.

A series of intensive searches were carried out of all accessible points of the Isle of May between 3^rd^ and 9^th^ December, and again on 11^th^ and 12^th^ January. All traumatically injured cases found during these searches are reported in Table 1.

### Description of pathology

The carcasses of 11 of the 14 traumatically injured pups found between the 28/11/2014 and 5/12/2014 were subjected to detailed necropsies. Three carcasses of seals killed on the 7^th^ and 8^th^ December could not be recovered but were examined on site for gross pathological features. All cases were scored using the scoring system used to identify corkscrew lesions in previous necropsies.

#### Gross Pathology

All fourteen cases had extensive traumatic lesions including large deep lacerations, detachment of skin and blubber layers and/or severe fractures to the front of the skull. All cases were in good physical condition with the significant blubber reserves expected in recently weaned pups. Gross examinations revealed no evidence for any underlying disease or disability and the pups were all assessed to have been healthy when the injuries were sustained. Four carcasses exhibited significant amounts of swallowed mud and fluid in the stomach and mud throughout the bronchial tree. Three carcasses had severe trauma to the front of the skull with facial bones fractured and significant haemorrhage into the sinuses and nasal turbinates, additionally a volume of blood appeared to been aspirated into the lungs. Five cases showed convincing evidence of hypovolemeia, notably pale musculature and light brown colouration to the liver due to blood loss. One case showed a bilateral pneumothorax with associated contraction of the lung. Based on the lesions, 7/11 (64%) cases primarily died from drowning and 4/11 (36%) from hypovolemic shock due to exsanguination. Specific gross pathological attributes are given in Table 1. The patterns of injuries, including apparent canine puncture wounds in the skull, and punctuate marks with associated haemorrhage through the pelt and into underlying tissue were compared to the dentition pattern from the skull of an adult male grey seal and were consistent with crushing bite wounds.

#### Histopathology

Histological examination was undertaken on lung tissue from two of the cases recovered from the freshwater pool and showed a number of emphysematous bullae in the alveolar regions directly adjacent to airways. There was debris within the airways comprising plant material, and silt or soil particles were observed in the larger airways. Given the absence of significant particulate matter in seawater and the rapid absorption of any aspirated water into the lung parenchyma, drowning could not however be excluded as the contributory factor in cases recovered from the tidal pools. Overall both gross and histopathology did not indicate any concurrent disease process which could plausibly indicate morbidity.

The most common morphological lesion identified were those consistent with predation, namely areas where significant portions of skin or tissue were missing (13/14 cases), teeth or claw rake marks in blubber (11/14 cases) and the undermining and separation of the blubber layer from the underlying fascia (12/14 cases). The characteristic smooth edged cut extending from the head caudally in a spiral around the body was seen in 9/14 cases, however of those, 5 cases also exhibited ragged edges to wound margins elsewhere on the animal. In all cases the removal of the blubber produced similar damage which, importantly, had previously been interpreted as post mortem scavenging of the carcass rather than a primary predation event.

The wound patterns were similar to those identified as corkscrew injuries in previous breeding seasons at the Isle of May and show very similar attributes to those observed in both grey seal pups and harbour seal adults necropsied in Norfolk, throughout Scotland and in Northern Ireland [3].

Although no individual case matched the ‘archetypal’ spiral lesion, there were significant and consistent similarities in pathological attributes (Table 1). At least 5 of the lesion attributes previously identified in corkscrew injuries were demonstrated in these cases from the Isle of May. Two ‘corkscrew’ attributes consistently not displayed were an absence of missing tissue and evidence of recent feeding, however the latter would be expected to be absent in weaned grey seal pups. Of the five cases observed to have been killed, two scored as typical corkscrew cases, two as similar and one as an atypical.

### Historical review of cases

Between 1985 and November 2015 149 dead seal cases were reported with unexplained trauma lesions in Scotland. Of these, 48 were recovered for necropsy, all since 2010. Retrospective assessment of the photographs and post mortem information for these cases was undertaken using the same criteria, with an attribute scoring of zero recorded for that animal if insufficient information was available to reliably confirm presence or absence of this attribute.

Table 2 shows the frequency of pathological attributes noted in all suspected spiral cases necropsied from the Scottish coast. The most common finding in spiral cases are, in decreasing order of frequency, missing tissue, undermining of blubber, avulsion of scapular and a smooth edge to the wound margin. These attributes are all consistent with the known grey seal predation cases observed on the Isle of May (Table 1).

**Table 2:** Attributes noted in all sprial trauma cases necropsied 2009-2015(n = 48).

	Number of cases with this attribute	Number of cases without this attribute	% of cases with attribute	Total assessable cases
**Areas of skin or tissue missing**	44	2	95.70%	46
**Undermining of blubber**	32	3	91.40%	35
**Avulsion of one or both scapula**	39	5	88.60%	44
**Smooth edged area to wound margin**	40	7	85.10%	47
**Rakemarks in blubber**	28	6	82.40%	34
**Single linear lesion (one or more rotations)**	38	10	79.20%	48
**Lesion begins at mouth**	31	12	72.10%	43
**Punctate lesions on muzzle**	24	12	66.70%	36
**No obvious tissue defects associated with wound margin**	21	15	58.30%	36
**Punctate lesions elsewhere**	21	17	55.30%	38
**Absence of associated skeletal trauma**	23	20	53.50%	43
**Ragged edged areas of wound margin**	21	24	46.70%	45
**Skeletal trauma to scapula**	19	24	44.20%	43
**Skeletal trauma noted to head**	18	27	40.00%	45

Of the historical cases, where the cause of death was not observed, 23 out of the 26 highest scoring corkscrew carcasses were found to have lesion attributes now likely to have been caused by seal predation (Table 3). Of the 48 trauma cases necropsied since 2010, 37 (77%) were either observed seal predation or showed pathology suggesting seal predation was highly likely. Additionally, 27/48 (56%) showed either archetypal or typical spiral lesions.

**Table 3:** Adjectival assessment of spiral seal cases.

Seal predation?	Archetypal	Typical	Similar	Atypical
**Definite, observed**	0	1	2	1
**Likely**	23	3	4	3
**Probable**	1	0	0	0
**Possible**	1	0	3	4
**Unknown**	1	1	0	0
**Unlikely**	0	0	0	0

## Discussion

On the basis of the physical characteristics of the lesions and the temporal and spatial distribution of carcass stranding sites previous studies have concluded that propeller interactions were the most likely cause of spiral lacerations on seal carcasses [1,2]. The observations reported here show that an adult male grey seal is capable of inflicting these types of injury through a process of biting and tearing the skin and blubber layer. The observations also show that a single male grey seal is capable of producing at least eight and possibly 14 corkscrew carcasses in ten days during the breeding season. The temporal pattern of strandings at the Isle of May could therefore be the result of the actions of a single adult male grey seal.

When tested against the standardised scoring system 12 out of the 14 pups scored in the top category for corkscrew injuries. This suggests that a proportion of the cases previously proposed as the result of interactions with propellers can be explained by grey seal predation. The locations of the carcasses on the Isle of May in 2014 were similar to those recorded in previous years and the injury patterns were similar. It is therefore highly likely that the same mechanism, i.e. grey seal predation is responsible for many or probably all of the recorded corkscrew mortalities identified at the Isle of May since 2010. It is possible that predation can account for many of the spiral lacerations seen in the rest of the UK and at various sites in Canada and Europe as grey seals are present in all areas where corkscrew wounds have been reported[3–5,16–19]. Conversely, in many places where we would expect ‘corkscrew’ seals if shipping interaction was the primary cause, there have been no reports of stranded animals with spiral lesions. In the north-eastern Pacific as an example, the absence of grey seals could explain the absence of corkscrew cases, despite the high concentration of shipping traffic in areas with large populations of harbour seals.

Grey seal predation could also explain the apparent seasonality observed in the historic cases. Grey seal pups with spiral lesions have been reported significantly more during winter months whereas harbour seal strandings bearing similar wounds appear almost exclusively during the spring and summer [2,3,5]. Concentrations of phocids at or close to haulout sites in the UK peak during breeding and moulting periods where the animals gather in localised areas and haul out for extended periods of time. In harbour seals the moult almost immediately follows the breeding season and the peak haulout counts are typically made between May-August [20,21]. Conversely, the grey seal breeding season in the UK varies greatly from site to site with the earliest pups born during October and the latest in early January [22]. The temporal distribution of corkscrew seal strandings appear to follow these seasonal patterns.

Preliminary attempts to reproduce the lesion patterns on carcases using clamps to mimic a predator’s jaws did not produce tearing wounds similar to the corkscrew wounds (Brownlow *unpublished data*). The skin and blubber layer eventually tore with the application of loads exceeding 220 kg but produced uneven tears dissimilar to the clean edged wounds seen in corkscrew cases. A series of scale model trials demonstrated that ducted propellers can produce these types of wounds [6]. Vessels with such mechanisms have been identified as potential causes in most of the observed events around Scotland. It would therefore be premature to assume that the interactions with propellers are not responsible for any of the observed injuries to seals. However, historical analysis of strandings coupled with direct observations of infanticide and cannibalism suggest that attacks by adult male grey seals could explain many if not all of the observed spiral lacerated seals in UK waters.

Pathology of retrieved carcasses indicated a traumatic cause of death in all 2014 Isle of May cases. While missing blubber was noted in 9 of the 11 necropsies, ante-mortem mass was not known and therefore only a qualitative assessment of the amount of missing tissue could be made. Previous reports of cannibalism in grey seals indicates significant proportions of flesh being removed from the carcasses [16,23]. Similarly Leopold et al. (2015) noted large quantities of blubber missing from harbour porpoises predated by grey seals and van Neer et al. (2014) noted large quantities of muscle and blubber absent from harbour seal carcasses observed being eaten by a grey seal. Video recording of the observed predation showed evidence of feeding on the carcasses however only relatively small amounts of blubber appeared missing from the carcasses examined at necropsy.

While feeding is by definition indicative of predation most predators will strive to gain the maximum amount of resources from a kill [24]. In this case the study male was observed to ingest only a small proportion of the blubber layer from each kill. In addition, he was witnessed killing a pup while the carcass of another, killed less than 24 hours earlier, lay beside him with a substantial proportion of blubber and muscle still intact. This may suggest further benefits of this behaviour in addition to resource acquisition. If energy reserves are a major limiting factor in the length of time a male can stay on the breeding colony, infanticide and cannibalism of pups should increase the male’s tenure which presumably could increase mating opportunities [16,25]. We don’t have a record of this male’s reproductive history and would require information on energy intake from cannibalism, its effects on length of stay and on mating opportunities to assess this potential benefit of cannibalism.

Surplus killing has been noted in many species including wolves [26] and owls [27] however in most cases there has been evidence of either the eventual intended consumption of that prey e.g. in food caching, or gaining experience in predatory tactics [28]. In this case the male was not displaying food caching behaviour as he did not consume any more of the prey after that initial feeding bout. Unexplained surplus killing of harbour seal pups has also been seen in killer whales (*Orcinus orca*) [29]. At present we do not know why this male seal ate so little of each carcass.

As a result of the observations, time of death was very accurately estimated (precisely measured in some cases). Carcasses were often retrieved within a few hours of death so the remaining blubber was unavailable to scavengers and showed very little autolysis. A previous study measuring the mean axial girth of a sample of live weaned pups at the Isle of May to be between 1995–1999 was 87.3 cm (StDev = 7.0cm) [30].The mean post mortem axial girth for the dead individuals observed in this study was 76.75 cm and only 4 of the 14 animals had axial girths above the mean value for live weaned pups. While ante-mortem girth measurements were not available, the girths of predated individuals are low suggesting a proportion of fat and muscle has been removed. As we do not have ante-mortem weights for any individuals we cannot make an accurate estimate of the blubber mass loss. It is therefore possible that higher proportions of blubber were removed than appeared during necropsy.

Grey seals are considered to be a predominantly piscivorous predator with the bulk of the diet consisting of benthic and demersal, fish and invertebrates [31–33]. Foraging behaviour typically consists of offshore trips range from local coastal sites to sites over a hundred of kilometres offshore with repeated dives to the seabed [33,34]. Investigations on the diet of grey seals which have focussed on hard part analyses of faecal samples were capable of identifying regional differences in type, proportions and seasonality of fish and invertebrate prey sources [31,32]. While no previous analyses has attempted to identify higher trophic level prey sources, diet structure and foraging behaviour may indicate the driver for the behaviours described in this study. However, some males have been observed adopting a semi-capital strategy to breeding, with individuals being observed making short foraging trips between bouts of copulation attempts [35,36]. In general, it is accepted that length of residence on the breeding site is related to number of copulation opportunities. Weaned and therefore undefended pups may represent a substantial and easily accessible energy source in their immediate vicinity. Eating pups may reduce the need for trips away from breeding sites and potentially increase the number copulation opportunities.

The striking difference in 2014 compared to previous years is the discovery of carcasses in freshwater pools above the tidal zone. Until 2014 all carcass locations at this site had been on beaches, in the sea or in tide pools. Of the 37 corkscrew carcasses on the Isle of May all but three had been reported in the water in or close to the tidal channel on the south east side of the island. The pups which were preyed upon were all healthy, recently weaned, naïve animals with a high lipid to lean mass ratio, typical of weaned phocid pups [37,38]. Adult females abandon their pups at weaning so they are not available to defend them from male aggression. Post-weaning fasts in grey seals are characterised by reduced metabolism and reduced movement [39] making the pups easy to approach and catch. An alternative strategy would therefore need to be adopted to catch pups in the water due to increased evasion potential by the prey. It is also likely that animals killed in the water, if not observed, would be discovered on average later than animals killed on land. If the actions of the study male in these observations are typical it would be safe to assume that the number of individuals with spiral lacerations found along the UK coast in recent years is a small fraction of the total number of animals predated by adult male grey seals.

Cannibalistic attacks by grey seal males on weaned pups in Wales have been observed regularly since 2009. The observed attacks were similar to those described here, but produced raged edged, more or less circular wounds on the back of the neck and shoulders[17,23]. The absence of the characteristic spiral lacerations in these and other observations of cannibalism may indicate that only a proportion of grey seal predation events will produce the characteristic corkscrew lesions.

Until now the phenomena of cannibalism has been considered infrequent and atypical of adult grey seal behaviour with few documented observations [7,16,17]. The observations presented here lead to the conclusion that many of the spiral laceration cases in the past may have been due to grey seal predation and may indicate that sympatric marine mammals have been an important but overlooked prey source for grey seals for many years. Quantifying the extent of such rarely observed events is difficult and we are therefore restricted to identifying cause of death and quantifying the extent of the injuries from post mortem data.

Previous studies have identified predatory species based on the presence of salivary DNA in wounds [40–43]. While capable of conclusively identifying predators, genetic differentiation to a species level is not possible in cases of conspecific aggression and cannibalism. Genetic analysis, to species level, of wounds on harbour seals would be feasible however the level of degradation is usually high and with the time of death uncertain, presence of grey seal DNA may only indicate scavenger damage rather than predation. Assessment of DNA from wounds to identify individual predators could be possible if carcasses are found reasonably soon after death. We suggest that all future non-grey seal casualties are subjected to genetic analysis, provided the level of autolysis and scavenger damage are reasonably low.

## Conclusion

In previous reports [2,3,6] it was argued convincingly that the wounds identified on grey seal pups and harbour seals in Scotland were the result of the same mechanism. The nature of the wounds, the stereotypical patterns of injuries and timing of events strongly suggests that a similar mechanism is responsible for the majority of reported corkscrew mortalities in the UK [3] and around the North Atlantic [4,5]. If the same argument holds here, the implication would be that a high proportion of the corkscrew injuries observed globally are likely to have been due to grey seal predation events and the population consequences of this behaviour may be significant. Predation on harbour seals and porpoises by grey seals demonstrates asymmetric intraguild predation whereby the predation event targets a species with which it competes for prey resources but also has value as a food resource. Understanding the prevalence, and potential drivers, of intraguild predation in these protected marine predators is an important next step to understand the potential ecological impact of these interactions.
